# Musical self-expression and empathy as dual pathways from group music participation to pro-social behavior: a structural equation modeling approach

**DOI:** 10.3389/fpsyg.2026.1744000

**Published:** 2026-03-18

**Authors:** Xiahong Qiao

**Affiliations:** Faculty of Arts, Ordos Institute of Technology, Ordos, China

**Keywords:** group music activity participation, musical empathy, musical self-expression, pro-social behavior, socio-emotional learning mechanisms

## Abstract

**Introduction:**

Pro-social behavior plays a crucial role in promoting individual social development and societal harmony. However, research suggests that pro-social behaviors among university students are not consistently enacted despite positive intentions. Group music participation offers a unique socio-emotional context that integrates emotional expression, interpersonal coordination, and shared experiences. Building on socio-emotional learning and self-determination theory, this study examines whether musical self-expression and musical empathy serve as mechanisms linking group music activity participation to pro-social behavior.

**Methods:**

Data were collected from Chinese university music students using a time-lagged survey design across three waves. A total of 387 valid responses were obtained from students enrolled in music-related programs at public universities in Inner Mongolia. Constructs including group music activity participation, musical self-expression, musical empathy, and pro-social behavior were measured using validated Likert-scale instruments. The proposed relationships and serial mediation model were analyzed using variance-based structural equation modeling (PLS-SEM).

**Results:**

The findings reveal a significant positive relationship between group music activity participation and pro-social behavior. Group music participation also positively predicts musical self-expression, which in turn enhances musical empathy. Both musical self-expression and musical empathy significantly mediate the relationship between group music activity participation and pro-social behavior. Furthermore, the results support a serial mediation pathway in which group music participation fosters musical self-expression, which subsequently promotes musical empathy and ultimately enhances pro-social behavior.

**Discussion:**

This study extends existing research by identifying socio-emotional learning mechanisms through which group music participation promotes pro-social development. The findings highlight the importance of emotional expression and empathic engagement in collaborative music-making contexts. These results provide theoretical contributions to music education and socio-emotional learning literature and offer practical implications for educators and institutions seeking to cultivate pro-social behaviors through group-based musical activities.

## 1 Introduction

Pro-social behavior refers to “all behaviors that are favorable to others and conducive to social harmony, such as helping, cooperating, sharing, and comforting” (Eisenberg, 2003; Wang et al., 2021, p. 1). According to preliminary research (e.g., Huang et al., 2023; Kong and Zeng, 2023; Yang et al., 2025; Zhang et al., 2022), pro-social behavior has a wide range of implications for both individual development and broader social outcomes. From an individual perspective, pro-social behavior can engender positive social adaptation, which serves as a significant stimulator of individual social adaptation (Getahun Abera, 2023; Monyei et al., 2022). On the other hand, social implications of pro-social behavior encapsulate but not limited to harmony, justice, and the development of the broader community (Xiao et al., 2023). Moreover, research also supports the notion that the implications of pro-social behavior go beyond individual and social outcomes and may also nourish emotional and psychological wellbeing of individuals (Man and Jing, 2025; Wan et al., 2023).

Despite these well-established benefits, recent studies conducted in Asian educational contexts—particularly in China—suggest that pro-social behaviors among university students are not consistently enacted in everyday situations (Xu et al., 2024; Yang et al., 2025). Although students often report altruistic intentions, their behavioral engagement in helping, sharing, or cooperation tends to be situational and short-lived (Wang et al., 2021). This intention–behavior gap highlights the need to examine not only whether pro-social tendencies exist, but also the socio-emotional mechanisms that sustain and translate social engagement into enduring pro-social behavior.

A review of literature on pro-sociality divulges that such behaviors don’t emerge in isolation, rather they require repeated social, emotional, and expressive interactions that allow individuals to experience and understand others’ perspectives (Kong and Zeng, 2023; Xu et al., 2024). Several theoretical perspectives suggest that pro-social behavior is shaped by collaborative processes (Getahun Abera, 2023; Xue et al., 2022), which induce a form of pro-social behavior (McGrath, 2025). Within this collaborative spectrum, music-making constitutes a particularly rich socio-emotional context, as it inherently integrates emotional expression, interpersonal coordination, and shared intentionality (Clark and Lonsdale, 2023). Despite its potential for cultivating pro-social dispositions, the specific emotional and interpersonal processes through which group music participation fosters pro-social behavior remain theoretically underdeveloped and empirically underexplored.

Accordingly, the present study examines the association between participation in group music activities and pro-social behavior within the context of Chinese university music students. Participation in music activities refers to “the process of individuals or groups interacting through different forms of music activity, covering a wide range of forms from individual activities to collective collaboration” (Yang et al., 2025; p. 3). Group music participation is not limited to joint task performance; rather, it involves rhythmic synchronization, embodied coordination, and shared affective experiences, all of which are central to socio-emotional learning and interpersonal attunement (Ilari et al., 2020; Pardo-Olmos et al., 2025).

In addition to examining the direct relationship between group music activity participation and pro-social behavior, the present study scaffolds within a socio-emotional learning framework and seeks to explore the underlying intrapersonal and interpersonal mechanisms that translate group music activity participation into amplified pro-social behaviors. Accordingly, the study answers the recent call from Yang et al. (2025) and proposes musical self-expression as an intrapersonal emotional process and musical empathy as a music-specific interpersonal socio-emotional process that operate within collective music-making contexts. Musical self-expression is conceptualized as the individual’s capacity to externalize, regulate, and communicate emotions through musical performance and interaction (von Moreau et al., 2010; Schäfer et al., 2013), whereas musical empathy refers to the ability to perceive, resonate with, and emotionally respond to others’ expressive states during shared musical activities (Rabinowitch et al., 2013; Davila-Barrio et al., 2023). This conceptualization distinguishes musical empathy from general dispositional empathy by situating it in embodied, temporally synchronized, and affectively shared musical experiences (Tzanaki, 2022; Morand-Grondin et al., 2026).

Underpinned in the self-determination theory (SDT) (Ryan and Deci, 2024), the study expects that group music activity participation may initially foster musical self-expression by supporting autonomy and competence needs through opportunities for creative contribution, interpretive choice, and perceived mastery in ensemble settings (MacIntyre et al., 2018; Sanguinetti, 2024). Importantly, the present study explicitly acknowledges that the proposed progression from musical self-expression to musical empathy represents a theoretically inferred pathway rather than a mechanism directly tested through the measurement of SDT’s basic psychological needs.

To strengthen this linkage, the serial mediation model is further informed by complementary socio-emotional and neurocognitive perspectives. Emotional contagion theory posits that expressed emotions—particularly in rhythmic, embodied, and socially shared contexts—can be transferred among individuals through automatic affective resonance (Hatfield et al., 1993; Tzanaki, 2022). Similarly, research on perceptual–motor coupling and the mirror neuron system suggests that synchronized musical actions and expressive gestures facilitate shared emotional understanding by aligning perception, action, and affect across co-participants (Overy and Molnar-Szakacs, 2009; Cross et al., 2012). Empirical evidence further demonstrates that collective music-making enhances empathic attunement through emotional synchronization and coordinated interaction (Wu and Lu, 2021; Cuervo and Campayo, 2024; Pardo-Olmos et al., 2025).

In nutshell, the study aims to extend the current line of inquiry on group music activity participation by assessing the serial mediation paths, leading to enhanced pro-social behavior. The study thus not only illuminates the emotional spectrum of music students but also highlights the social dimension of their experiences, which may carry far-reaching implications both for personal development and collective harmony.

The remainder of the study presents theoretical framework and hypotheses development, followed by deliberating research design and methodology, discussing study’s findings, and offering theoretical as well as practical implications.

## 2 Theoretical framework and hypotheses development

### 2.1 Linking group music activity participation with pro-social behavior

Ethnographic studies and case studies provide a nexus between participation in group music activities and social and pro-social skills and community building (Ilari et al., 2020; Polzella and Foris, 2014; Ryan and Deci, 2024). The findings from those preliminary studies provoke the idea that participation in music-making activities may nurture group affiliation through one’s sense of belonging and cooperation, potentially translating into enhanced pro-social development (Clark and Giacomantonio, 2013; Yang et al., 2025). In this perspective, one of the key features of group music activities involves the interpersonal movement synchronization, which advances interpersonal similitude and coordination (Pardo-Olmos et al., 2025). Moreover, group music activities allow individuals to nurture interpersonal communication; they modulate mood and emotions, while preserving the semantic ambiguity of individuals (Ruth, 2018). That is to say, collective music-making gives a sense of autonomy to individuals while at the same time remaining engaged in the collective expression. In addition, individuals can engage in imitation and mimesis as group music activities provide a safe space, resulting in increased sense of togetherness, which in turn, promotes pro-social behavior. This is further supported by Moore et al. (2023), who corroborated that affiliation is the key recipe for pro-social behavior among children as well as adults, including in musical contexts (Ilari et al., 2020). From a coordination-based perspective, synchronized rhythmic engagement fosters shared intentionality and collective identity, both of which have been empirically linked to cooperative and helping tendencies (Kirschner and Tomasello, 2010; Wiltermuth and Heath, 2009). Hence, the study proposes its first hypothesis as:

*H1:* Participation in group music activity has a positive relationship with pro-social behavior among music students.

### 2.2 Linking group music activity participation with musical self-expression

Prior research indicates that music has many gratifications and uses (Huang et al., 2021; Sedikides et al., 2022). Rooted in classical ethnomusicological literature, Merriam (1964) posited 10 key social functions of music: symbolic representation, communication, entertainment, aesthetic enjoyment, emotional expression, the stability and continuity of culture, enforcing conformity to social norms, the integration of society, and validating religious rituals and social institutions. According to subsequent literature Schäfer et al. (2013), the operationalization of these functions occurs at multiple levels. For instance, at the individual level, music may evoke physiological responses such as arousal or relaxation and serve as a means of emotional regulation. At the group level, collective musical experiences foster shared understanding and collaboration, which can cultivate social connectedness and sense of belonging (Clare and Camic, 2020). Such participatory activities allow individuals to explore and express their identities through sound and interaction (Clark and Lonsdale, 2023). Therefore, the study anticipates that participation in group musical activity can be positively associated with musical self-expression.

This notion is further supported by Kiss and Linnell (2023), who found that participation in musical activities fosters the development of personal identity and agency while simultaneously promoting mood regulation and relaxation. In a diverse context, for example, in the participatory sport theater project; Kettunen et al. (2021) found that collaborative activities helped the participants promote a sense of inclusion, identify new personal strengths, and nurture a new form of self-expression. Moreover, this assumption is underpinned in the SDT’s first two dimensions, *namely*, autonomy and competence (Ryan and Deci, 2024). Group music activity participation allows individuals choose how to interpret, improvise, or contribute within the music-making process, which in turn, nurtures individuals’ perceptions of autonomy and competence as they collaborate, perform, and contribute meaningfully to the collective musical experience.

Beyond the direct link, the study further predicts that musical self-expression may also function as an intrapersonal mechanism through which participation in group music activity translates into enhanced pro-social behavior. A review of research on individual psychological predispositions reveals that the feelings of being controlled or obligated by external contingencies negatively relate to pro-social behavior (Chen, 2025; Kındap-Tepe and Aktaş, 2021). This is because individual differences and environmental forces play role in the execution of volunteering actions (Thielmann et al., 2020). On the other hand, human tendency to exhibit pro-social behavior largely depend on an individual’s internal motivational drive, which directly fosters greater engagement in helping others. Anchored in the SDT theory (Ryan and Deci, 2024), the study expects that participation in group music activity leads to nourishing increased self-expression, which in turn, leads to improved pro-social behavior. Expressive authenticity may strengthen internalized motivation, thereby increasing the likelihood of voluntary helping behaviors emerging from self-endorsed values rather than external pressure. According to Yang et al. (2025), group interaction, i.e., participation in group music activities, and emotional synchronization, i.e., musical self-expression, generate the feelings of belongingness among participants. This is further supported by Tzanaki (2022), who contended that the feelings of trust and belongingness among participants can be spurred through even simple rhythmic activities performed in synchrony, which in turn, serves as a bridge to promote pro-social behavior among participants. Therefore, the study proposes:

*H2:* Participation in group music activity has a positive relationship with musical self-expression.

*H3:* Musical self-expression mediates the relationship between group music activity participation and pro-social behavior.

### 2.3 Linking musical self-expression with musical empathy

Empathy represents a complex and multidimensional phenomenon that continues to challenge scholars across psychology, education, and neuroscience (Goleman and Boyatzis, 2017). Following its seminal inclusion in Daniel Goleman’s conceptualization of emotional intelligence, empathy has taken a central role in contemporary socio-emotional research (Goleman, 2001), particularly within the domain of music-making (Tzanaki, 2022). According to scholars (e.g., Davila-Barrio et al., 2023; Morand-Grondin et al., 2026), empathy has two distinctive perspectives: *cognitive* and *affective*. The cognitive perspective involves “the imagining and understanding of what another person is feeling by taking their perspective–the so-called cognitive empathy” (p. 2). On the other hand, the affective perspective relies on the premise of emotional contagion to understand other person’s perspective–i.e., “feeling what the other person is experiencing” (p. 2).

Given that music is inherently an emotional experience, the study further predicts that musical self-expression will be positively associated with musical empathy. This argument is laid down on the SDT theory’s relatedness dimension (Ryan and Deci, 2024). According to this theory MacIntyre et al. (2018), when autonomy and competence needs are fulfilled, for instance, in the form of musical self-expression, individuals strive to strive accomplishing their relatedness need. That is to say, group music activity participation lays the foundation for fostering participants self-expression by satisfying the psychological needs for autonomy and competence through the sense of mastery and effectiveness within the collective musical experiences (Sanguinetti, 2024). Once autonomy and competence needs are satisfied, participants naturally enter an emotional state of connectedness, wherein they begin to perceive and interpret others’ perspectives through the collaborative lens of music-making.

This assumption can be further explained through the perceptual-motor coordination approach (Overy and Molnar-Szakacs, 2009), which posits that individual’s body movement and limb coordination and synchronization are tied to periodic environmental events via auditory, haptic, or visual information. In a similar vein, Cross et al. (2012) found that anticipation and self-perception skills are guided by interactive group behavior, which, in turn, facilitates individuals to experience similar feelings and thoughts for others. Marin and Gingras (2024) also acknowledged this notion that music has the power of affecting emotional state and behavior of human beings. In this perspective, Jeremić et al. (2015) conducted an experimental study on two groups of music students. The authors experimented learning methods for vocal performance based on learning songs by ear. Participants those in the experimental group showed incremental socio-emotional skills as compared to those in the controlled group. Subsequent findings in previous research also yield that music-making inducing emotions in participants influences listeners’ emotional and social behavior (Morand-Grondin et al., 2026; Wu and Lu, 2021). Collectively, these findings suggest that expressive engagement may heighten sensitivity to others’ emotional signals, thereby facilitating empathic attunement during joint performance. Taken together, the study anticipates that musical self-expression and musical empathy are the serial mediating mechanisms that link group music activity participation and pro-social behavior. Therefore, the study proposes the next hypotheses as follows:

*H4:* Musical self-expression positively predicts musical empathy.

*H5:* Musical empathy mediates the relationship between musical self-expression and pro-social behavior.

**FIGURE 1:**
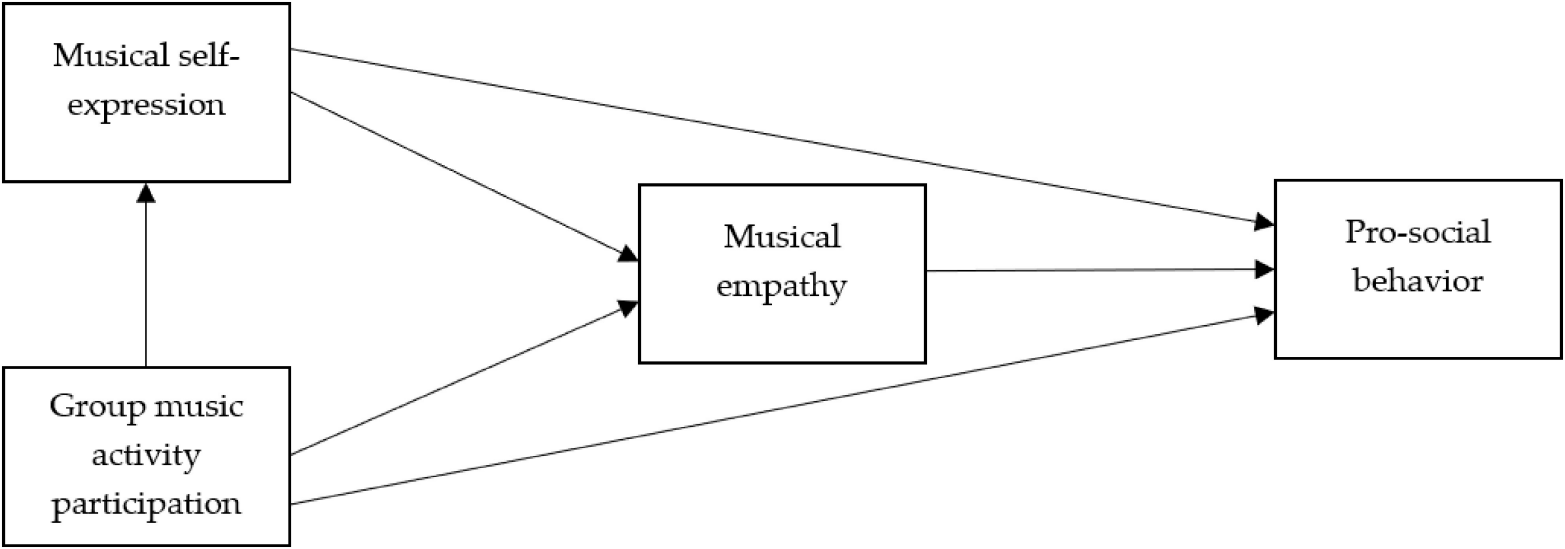
Conceptual model.

*H6:* Musical self-expression and musical empathy sequentially mediate the relationship between group music activity participation and pro-social behavior (Figure 1).

## 3 Research methodology

### 3.1 Research design

The study is conducted to investigate the indirect effect of group music activity participation on music students’ pro-social behavior through the serial mediating roles of musical self-expression and musical empathy. For this reason, the study is designed to empirically analyze data gathered from Chinese university music students using the survey technique. In addition, given the correlational research design, the study employs a time-lagged data collection technique to examine the mediation paths. This technique is used to minimize common method biasness/monomethod issues pertaining to the cross-sectional research design (Law et al., 2016).

Further, a purposive sampling technique is used in this study to collect data from Chinese university music students. This technique is suitable for its flexibility and ease, while also yielding arbitrary responses which are crucial for the non-probability sampling technique. Chinese university music students are approached and given questionnaires along with cover letters, specifying study’s objectives, ethical compliance, and keys for generating responses.

Data were collected from music students enrolled in three public universities located in Inner Mongolia, China. Access to participants was facilitated through departmental coordinators, and questionnaires were distributed during scheduled class sessions. Participation was voluntary and anonymous. To ensure longitudinal matching across waves, respondents were instructed to generate a confidential identification code based on non-identifiable personal cues, which enabled accurate pairing of responses across the three waves while preserving anonymity. Unmatched or incomplete responses were excluded from final analysis.

A three-wave research design is employed with each wave collecting data from the music students on demographic profiles, group music participation, and pro-social behavior (wave 1), musical self-expression (wave 2), and pro-social behavior (wave 3). Total 500 responses were distributed, keeping in mind the decline in the response rate in a time-lagged study, and minimum appropriate sample size for SmartPLS SEM studies (Hair et al., 2019). First wave generated a response of 453, followed by the second wave with a response of 419, and finally yielding 387 responses after eliminating all the inaccurate/mismatched responses.

The final matched sample represents a retention rate of 85.4% from Wave 1 to Wave 3. Missing data were minimal (< 3%) and handled using mean substitution when necessary. Multivariate outliers were assessed using Mahalanobis distance and standardized z-scores (± 3.29), and no cases exceeded critical thresholds.

Of 387, 54% represented female and 46% reflected male music students, with an average age of 21 (SD: 2.47). Of total, 23% were in their first year, 31% were in second year, 28% were in the third year, and 18% were in their final year degree programs. Regarding major discipline, 36% were in music performance, 32% were in music education, 17% are in composition, and 15% are in music technology. Type of musical activity includes 35% representing vocal ensemble, 42% reflecting instrumental ensemble, and 23% were mixed.

### 3.2 Research instruments

The research instruments for this study are inspired from the previous studies and adapted within our study’s context. All the scale items are back-translated into Mandarin and are asked using a five-point Likert scale. Specifically, a standard translation–back-translation procedure was employed, whereby the original English items were translated into Mandarin by a bilingual academic expert and subsequently back-translated into English by an independent translator to ensure semantic equivalence. Minor wording adjustments were made to align items with the higher education music context in China. All constructs were modeled as reflective latent variables, and their factor structure and psychometric properties were assessed using PLS-SEM measurement model evaluation procedures. The full list of scale items is provided in Appendix 1 to enhance transparency.

#### 3.2.1 Group music activity participation

For measuring group music activity participation, the scale items are adapted from Fredricks et al. (2004); Yang et al. (2025), which consisted of four items. For example, “I work with my classmates to ensure that music activities go smoothly.” The construct captures collaborative engagement in ensemble-based music contexts and was validated through confirmatory measurement model assessment, demonstrating satisfactory reliability and convergent validity within the Chinese university setting.

#### 3.2.2 Musical self-expression

For analyzing musical self-expression, the scale items are adapted from von Moreau et al. (2010), which consisted of four items. For example, “I use music to communicate feelings that cannot be expressed verbally.” The items assess the extent to which students perceive music as a medium for emotional articulation and identity-relevant expression. The adapted scale retained its unidimensional structure in the present sample, with all indicators loading significantly on the intended latent construct.

#### 3.2.3 Musical empathy

For measuring musical empathy, the scale items are adapted from Rabinowitch et al.’s (2013) musical empathy scale, which consisted of four items. For example, “I feel emotionally connected to others while we play or sing together.” This scale operationalizes empathy within the specific context of joint music performance rather than general dispositional empathy. Measurement validation confirmed acceptable reliability and discriminant validity in the Chinese higher education context.

#### 3.2.4 Pro-social behavior

For assessing pro-social behavior, the scale items are adapted from Carlo and Randall’s (2002) pro-social tendency measure; operationalized in Yang et al. (2025), which consisted of four items. For example, “In an emergency, I don’t hesitate to help.” The scale captures behavioral tendencies across helping, sharing, and cooperative dimensions. Both Time 1 and Time 3 measurements employed identical items to ensure comparability across waves. The construct demonstrated stable factor structure and satisfactory psychometric properties across both measurement occasions.

### 3.3 Ethical considerations

This research complied with the ethical standards specified in the Declaration of Helsinki and observed all applicable institutional and national research ethics regulations. Before data collection, ethical approval was obtained from the Institutional Review Board of Ordos Institute of Technology. Involvement in the study was completely voluntary, and all participants were made aware of the research goals, methods, and their rights as subjects. Informed consent in writing was acquired from every participant prior to the start of data collection. To maintain participant anonymity and confidentiality, no personal information was gathered, and all replies were assigned numerical codes. The gathered information was safely stored in password-protected files that only the research team could access. Participants were informed that their answers would be used exclusively for research purposes and that they could exit the study at any point without facing any negative repercussions.

## 4 Results

The findings of this study are obtained by analyzing the survey data using SmartPLS SEM approach. The main reason to employ SmartPLS SEM is the complexity in the research design, encompassing serial mediation intrapersonal and interpersonal mechanisms (Hair et al., 2019). Furthermore, PLS SEM approach is an advanced multivariate data analytical technique that is capable of estimating the predictive capability of the hypothesized model (Hair et al., 2019). The analytical tool analyzes the survey data in two stages. In the first step, the outer model is estimated and the subsequent analysis is conducted on the inner model.

Given the time-lagged design and the collection of pro-social behavior at both Wave 1 (T1) and Wave 3 (T3), pro-social behavior at T1 was included as a control variable predicting pro-social behavior at T3 to account for baseline levels and examine change over time.

Prior to running the PLS algorithm, the study assesses the common method biasness (CMB) issues using the Harman’s one factor test. The test result indicated the first factor accounted for 32.45% of the total variance, confirming the absence of CMB issues in our study. In addition to Harman’s single-factor test, a full collinearity assessment was conducted using variance inflation factor (VIF) values as recommended in contemporary PLS-SEM literature. All latent construct VIF values were below the conservative threshold of 3.3, indicating that common method bias is unlikely to threaten the validity of the results.

After successful estimation of the Harman’s single factor analysis, the study assesses the measurement model to assure indicator and constructs’ reliability and validity. Results of this analysis are reported in Table 1, which show that most the outer loadings values surpass the minimum threshold value of 0.7 and that one indicator (GMAP1) is marginally below 0.708 but remains above the acceptable 0.6 cutoff (Hair et al., 2019). Specifically, GMAP1 exhibited an outer loading of 0.685; however, it was retained because the construct-level reliability and convergent validity remained satisfactory (CA = 0.769, CR = 0.852, AVE = 0.592), and removing GMAP1 did not yield a substantive improvement in CR or AVE. This analysis confirms indicators’ reliability in our study. Pertaining to the constructs’ reliability and validity, Cronbach’s alpha (CA), composite reliability (CR), and average variance extracted (AVE) are examined. Results reported in Table 1 provide methodological support for the reliability (i.e., value > 0.7) and convergent validity (i.e., value > 0.5) of the hypothesized constructs (Hair et al., 2019).

**TABLE 1:** Indicators and constructs’ reliability and validity.

Variables	Group music activity participation	Musical empathy	Musical self-expression	Pro-social behavior (T1)	Pro-social behavior (T3)	CA	CR	AVE
Group music activity participation						0.769	0.852	0.592
GMAP1	0.685							
GMAP2	0.815							
GMAP3	0.782							
GMAP4	0.789							
Musical empathy						0.833	0.888	0.665
ME1		0.804						
ME2		0.824						
ME3		0.837						
ME4		0.796						
Musical self-expression						0.805	0.873	0.632
MSE1			0.752					
MSE2			0.780					
MSE3			0.782					
MSE4			0.861					
Pro-social behavior (T1)						0.859	0.906	0.707
PSB1_T1				0.891				
PSB2_T1				0.873				
PSB3_T1				0.880				
PSB4_T1				0.706				
Pro-social behavior (T3)						0.828	0.885	0.659
PSB1_T3					0.764			
PSB2_T3					0.842			
PSB3_T3					0.816			
PSB4_T3					0.823			

According to Hair et al. (2019), the researchers should examine the discriminant validity of the chosen constructs, post successful evaluation of the convergent validity. For that reason, the Fornell-Larcker criterion and heterotrait-monotrait (HTMT) ratio are used. The Fornell-Larcker test compares the square root of the AVE with its correlation with other constructs. Values reported in the diagonal represent the square root of AVE, which are higher than their correlation values with other constructs, establishing discriminant validity. In addition, the HTMT ratio is estimated using the bias-corrected and accelerated (BCa) bootstrapping technique. Following recent PLS-SEM recommendations, discriminant validity is established when HTMT values remain below the conservative threshold of 0.90 and when bootstrapped confidence intervals do not include 1. The results presented in Table 2 show that all the HTMT values are lesser than 0.90, including the correlation between pro-social behavior at Time 1 and Time 3, further supporting the discriminant validity assessment.

**TABLE 2:** Discriminant validity using Fornell-Larcker and HTMT ratio.

Fornell-Larcker						HTMT ratio					
Variables	1	2	3	4	5	Variables	1	2	3	4	5
1. Group music activity participation	0.769	0.815	0.795	0.841		1. Group music activity participation	0.629	0.593	0.676	0.696	
2. Musical empathy	0.532					2. Musical empathy					
3. Musical self-expression	0.417	0.490				3. Musical self-expression	0.511				
4. Pro-social behavior (T1)	0.542	0.669	0.566			4. Pro-social behavior (T1)	0.641	0.786			
5. Pro-social behavior (T3)	0.694	0.657	0.622	0.593	0.812	5. Pro-social behavior (T3)	0.857	0.767	0.754		

The measurement model is further examined using the variance inflation factor (VIF), which is used to assess the multicollinearity issues in the study. Results shown in Table 3 indicate that all the VIF values are lesser than 3.3, which confirms that the study is free from the CMB issues. Following contemporary PLS-SEM recommendations, the VIF values were examined under the full collinearity assessment framework, where values below the conservative threshold of 3.3 indicate that common method bias is unlikely to contaminate the structural relationships. All latent construct VIF values were below this threshold, providing robust evidence that multicollinearity and common method bias are not major concerns in the present study. Accordingly, the full collinearity assessment serves as the primary statistical test for CMB in this research design.

**TABLE 3:** Variance inflation factor (multicollinearity assessment).

Variables	Group music activity participation	Musical empathy	Musical self-efficacy	Pro-social behavior (T1)	Pro-social behavior (T3)
Group music activity participation		1.210	1.000		1.549
Musical empathy					1.997
Musical self-expression		1.210			1.542
Pro-social behavior (T1)					2.219
Pro-social behavior (T3)					

**FIGURE 2:**
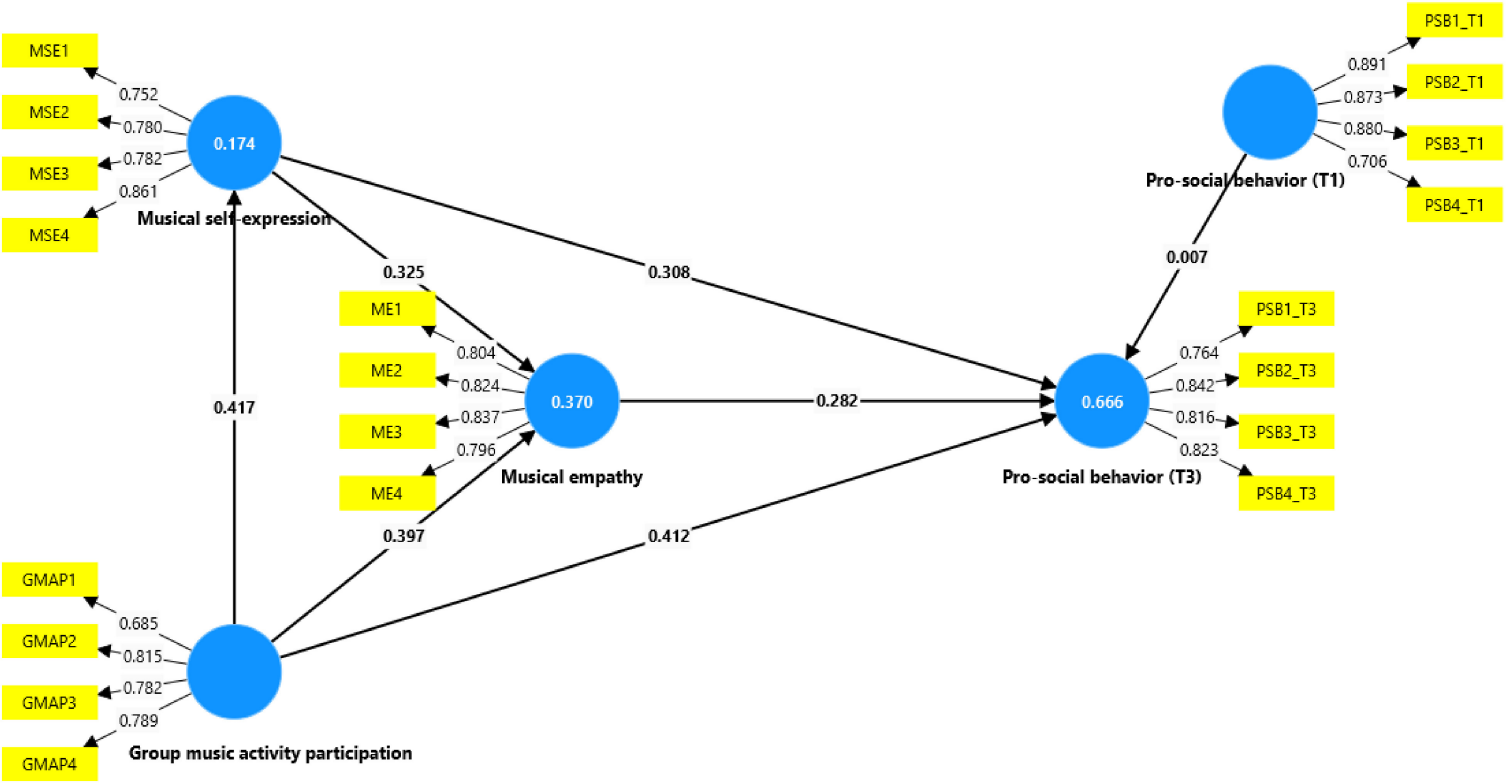
Structural equation model.

The structural model is evaluated to measure the structural paths and predictive capability, as shown in Figure 2 and Table 4. These results reveal that group music activity participation has a significant positive association with music students’ pro-social behavior at Time 3 [β = 0.412; *p* = 0.000; *t* = 6.287; 95% CI (0.283, 0.554)], supporting hypothesis 1. As hypothesized above, the study also examines the indirect relationship between group music activity participation and pro-social behavior through intrapersonal emotional and interpersonal socio-emotional mechanisms. Accordingly, the direct relationship between group music activity participation and musical self-expression is also significant and positive [β = 0.417; *p* = 0.000; *t* = 7.634; 95% CI (0.307, 0.517)], supporting hypothesis 2. Moreover, the study finds a significant positive association between musical self-expression and musical empathy [β = 0.325; *p* = 0.000; *t* = 4.716; 95% CI (0.177, 0.456)], supporting hypothesis 4.

**TABLE 4:** Hypotheses testing.

Relationship	Path	Confidence intervals	S.E.	T	P	Decision
Group music activity participation—> pro-social behavior (T3) (*H1*)	0.412	0.283, 0.554	0.065	6.287	0.000	Accept
Group music activity participation—> musical self-expression (*H2*)	0.417	0.307, 0.517	0.055	7.634	0.000	Accept
Group music activity participation—> musical self-expression—> pro-social behavior (T3) (*H3*)	0.128	0.077, 0.186	0.028	4.543	0.000	Accept
Musical self-expression—> musical empathy (*H4*)	0.325	0.177, 0.456	0.069	4.716	0.000	Accept
Musical self-expression—> musical empathy—> pro-social behavior (T3) (*H5*)	0.092	0.039, 0.156	0.030	3.027	0.001	Accept
Group music activity participation—> musical self-expression—> musical empathy—> pro-social behavior (T3) (*H6*)	0.038	0.017, 0.066	0.013	3.043	0.000	Accept
Pro-social behavior (T1) —> pro-social behavior (T3)	0.007	−0.134, 0.143	0.071	0.093	0.926	Not significant (control variable)

*p* < 0.05.

As hypothesized, the study finds significant mediating role of musical self-expression between group music activity participation and pro-social behavior [β = 0.128; *p* = 0.000; *t* = 4.543; 95% CI (0.077, 0.186)], supporting hypothesis 3. Similarly, musical empathy’s mediating role between musical self-expression and pro-social behavior is also significant and positive [β = 0.092; *p* = 0.001; *t* = 3.027; 95% CI (0.039, 0.156)], supporting hypothesis 5. Finally, the study finds that both musical self-expression and musical empathy significantly serially mediate the indirect relationship between group music activity participation and pro-social behavior [β = 0.038; *p* = 0.000; *t* = 3.043; 95% CI (0.017, 0.066)], supporting hypothesis 6.

Importantly, pro-social behavior at Time 1 was included as a control variable predicting pro-social behavior at Time 3 to account for baseline levels. The control path was non-significant [β = 0.007; *p* = 0.926; 95% CI (−0.134, 0.143)], indicating that the effects observed at Time 3 are not merely a continuation of prior pro-social tendencies. This strengthens the temporal inference that group music participation and the associated socio-emotional mechanisms contribute to changes in pro-social behavior over time.

Taken together, these analyses render support to our theoretical underpinnings that group music activity participation prompts intrapersonal emotional mechanism in individuals, which is further translated into interpersonal socio-emotional mechanism, finally culminating into improved pro-social outcomes over time.

To further evaluate the model’s explanatory and predictive capability, the coefficient of determination (*R*^2^), predictive relevance (*Q*^2^), and effect size (*f*^2^) were examined, as presented in Tables 5, 6. The *R*^2^ values indicate that the model explains 17.4% of the variance in musical self-expression, 37.0% in musical empathy, and 66.6% in pro-social behavior at Time 3. According to established guidelines, these values reflect weak-to-moderate explanatory power for musical self-expression, moderate explanatory power for musical empathy, and substantial explanatory power for pro-social behavior at Time 3.

**TABLE 5:** Coefficient of determination (R-square) and blindfolding (q-square).

Variables	R-square	Q-square
Musical empathy	0.370	0.235
Musical self-expression	0.174	0.107
Pro-social behavior (T3)	0.666	0.425

**TABLE 6:** Effect size (f-square).

Variables	Group music activity participation	Musical empathy	Musical self-expression	Pro-social behavior (T1)	Pro-social behavior (T3)
Group music activity participation		0.207	0.210		0.328
Musical empathy					0.120
Musical self-expression		0.138			0.184
Pro-social behavior (T1)					0.000
Pro-social behavior (T3)					

Blindfolding results further demonstrate predictive relevance, as all *Q*^2^ values are > 0 (*Q*^2^ = 0.107 for musical self-expression; *Q*^2^ = 0.235 for musical empathy; *Q*^2^ = 0.425 for pro-social behavior at Time 3). This confirms that the structural model possesses meaningful predictive capability for new observations.

Effect size (*f*^2^) analysis indicates that group music activity participation exerts a moderate effect on musical self-expression (*f*^2^ = 0.210) and pro-social behavior at Time 3 (*f*^2^ = 0.328), while musical self-expression and musical empathy contribute small-to-moderate effects to downstream constructs (*f*^2^ ranging from 0.120 to 0.184). In contrast, the effect size of pro-social behavior at Time 1 on pro-social behavior at Time 3 is negligible (*f*^2^ = 0.000), reinforcing the non-significant control path reported earlier.

## 5 Discussion and conclusion

The main objective in this study is to examine the intra-personal emotional and inter-personal socio-emotional mediating mechanisms that shape group music activity participation in enhanced pro-social behaviors. For this reason, the study analyzes time-lagged data gathered from Chinese university music students using multivariate analytical techniques, while controlling for baseline pro-social behavior at Time 1 to strengthen temporal inference. The results provide empirical support for the proposed serial mediation model, demonstrating that group music activity participation significantly influences pro-social behavior at Time 3 through the sequential mechanisms of musical self-expression and musical empathy. The findings below present unique and noteworthy implications for both theory and practice.

### 5.1 Theoretical implications

The findings of this study contribute to the music education, music students’ psychological predispositions, and pro-sociality literature in three major ways. First, these findings extend our understanding of how group music activity participation relates to intra-personal emotional process through an SDT perspective. Drawing on self-determination theory (SDT) (Ryan and Deci, 2024), the study illuminates the first two dimensions of SDT—autonomy and competence—as the overarching psychological conditions that help transform collective music experiences into identity-relevant self-expression. The study thus corroborates that group music activity participation not only shapes collaborative experiences; nonetheless, participants’ individual selves are nurtured through shared performance contexts. This is because when musicians perform in groups, they must synchronize rhythm, timing, and expressive cues, which enhances both perceived agency and mastery (Kirschner and Tomasello, 2010; Overy and Molnar-Szakacs, 2009). Such coordinated musical engagement has been shown to heighten feelings of competence and intrinsic motivation, which are central to expressive self-development (Ryan and Deci, 2024; Krause et al., 2019). Previous findings have assessed group music activity through a social identity perspective (Yang et al., 2025), social cognitive theory (Fleszar-Pavlovic et al., 2025), and theory of planned behavior (Yoo, 2020), or examined its association with wellbeing (Krause et al., 2019) and quality of life (McCrary et al., 2022). By linking collective music participation to self-expression through motivational and socio-cognitive lenses, the present findings integrate these strands of literature into a process-based explanation of pro-social development.

Second, by introducing musical empathy as the inter-personal socio-emotional pathway, this research advances understanding within music education and socio-psychological scholarship. Empirical evidence suggests that synchronized music-making enhances sensitivity to others’ emotional states and facilitates perspective-taking (Rabinowitch et al., 2013; Davila-Barrio et al., 2023). The SDT framework (Ryan and Deci, 2024) posits that satisfaction of autonomy and competence needs may increase the pursuit of relatedness, which is reflected in greater empathic engagement during collaborative activity. This interpretation aligns with emotional contagion theory (Hatfield et al., 1993), which proposes that expressive behaviors can elicit shared affective responses in interactive contexts, and with neurocognitive accounts emphasizing perception–action coupling in joint musical performance (Overy and Molnar-Szakacs, 2009; Cross et al., 2012). Prior research supports the notion that collective music-making fosters empathetic understanding through shared affect and mutual responsiveness (Cuervo and Campayo, 2024). Furthermore, empathy research within emotional intelligence scholarship underscores that domain-specific interactive practices—such as ensemble music—can strengthen empathic competencies through repeated affective exchange (Goleman and Boyatzis, 2017; Rabinowitch et al., 2013). The present findings therefore situate musical empathy within broader affective and motivational frameworks.

Last but not the least, these findings address the intra-personal emotional and interpersonal socio-emotional mechanisms mediating the relationship between group music activity participation and pro-social behavior. To the best of current knowledge, this is among the first empirical studies examining musical self-expression and musical empathy as sequential mediators linking collective music engagement to pro-social outcomes. Prior work has highlighted social connectedness and peer support as mediating mechanisms (Yang et al., 2025), whereas the present findings emphasize internal emotional transformation and interpersonal attunement processes. This process-oriented explanation is consistent with evidence that synchronized cooperative activities increase affiliative tendencies and helping behaviors (Kirschner and Tomasello, 2010; Wiltermuth and Heath, 2009), with music providing a uniquely emotion-laden medium for such coordination (Rabinowitch et al., 2013; Davila-Barrio et al., 2023). By integrating motivational theory (Ryan and Deci, 2024), socio-cognitive perspectives (Fleszar-Pavlovic et al., 2025), and affective synchronization research (Hatfield et al., 1993), the study advances a multi-layered understanding of how collective musical experiences translate into pro-social development.

### 5.2 Practical implications

The findings offer several actionable implications for music educators, curriculum designers, and institutional policymakers. First, given that musical self-expression emerged as the initial and foundational mechanism in the serial pathway, instructional design should prioritize structured opportunities for individual expressive engagement before emphasizing complex interpersonal coordination. For example, ensemble sessions may begin with short improvisational segments, solo interpretative passages, or reflective expressive exercises that allow students to experience autonomy and competence in performance. Establishing this foundation may enhance students’ expressive confidence, which subsequently facilitates empathic engagement within the group context.

Second, music curricula may be sequenced developmentally to mirror the serial mechanism identified in this study. Early stage ensemble training can focus on expressive authenticity and interpretive ownership, while later stages progressively integrate synchronized tasks requiring heightened interpersonal responsiveness, such as call-and-response exercises, dynamic phrasing alignment, and emotional mirroring practices. This staged approach ensures that intrapersonal emotional grounding precedes and supports interpersonal socio-emotional coordination.

Third, teacher training programs should incorporate pedagogical modules that help instructors recognize and intentionally cultivate expressive-empathic transitions in classroom practice. Educators can be trained to provide feedback not only on technical accuracy but also on emotional articulation and relational attunement within ensemble settings. By explicitly guiding students from expressive clarity toward empathic synchronization, instructors can strengthen the psychological processes that ultimately foster pro-social tendencies.

Fourth, institutions designing music-based interventions aimed at social development should structure programs that intentionally combine expressive creativity with synchronized collaboration. Community music initiatives, university ensemble programs, and arts-based social cohesion projects may benefit from incorporating phased models that move from individual voice exploration to collective emotional alignment.

Taken together, these recommendations translate the empirically supported serial model into pedagogically structured strategies that can enhance both musical development and pro-social outcomes in higher education contexts.

### 5.3 Limitations

Despite the significant findings of this study, the study is not free from its limitations. The findings thus should be interpreted with caution. First, although the study employed a time-lagged design to strengthen temporal ordering, the research remains correlational in nature and does not establish definitive causality. Moreover, the use of a self-report survey design may introduce response-related biases, including social desirability bias and common rater effects, which could potentially inflate associations among constructs. Although the study addresses the CMB issues empirically and by using a time-lagged research design, future studies should overcome this limitation by utilizing a multi-source, behavioral, or experimental research design to enhance causal inference and reduce method bias.

Second, the data used in this study is gathered by surveying Chinese university music education students. The purposive (non-probability) sampling approach limits statistical generalizability beyond the sampled population. Hence, the findings shall not be extended to the Western contexts and may, therefore, require replication across various cultural settings, age groups, and academic disciplines. Given the culturally specific and domain-specific nature of the sample, caution must be exercised in generalizing these findings to broader student populations or non-music educational environments. Future research is encouraged to replicate the proposed model across diverse cultural contexts, institutional types, and non-music collaborative domains.

Third, the study examined the mediating roles of intrapersonal emotional and interpersonal socio-emotional perspectives; however, overlooked the necessary boundary conditions that may be taken into account in future studies. Future research should use institutional support and individual factors, such as personality traits, as boundary conditions facilitating this phenomenon. Incorporating moderators such as cultural orientation, prior musical training intensity, or personality dimensions (e.g., openness to experience) may provide a more nuanced understanding of when and for whom the proposed mechanism operates most strongly.

Last but not least, future research can be carried out on professional musicians utilizing the proposed theoretical framework to understand contextual differences between students and professionals in exhibiting pro-social behaviors. Comparative studies between amateur, academic, and professional ensembles may further clarify the developmental and contextual robustness of the serial mechanism identified in this study.

## Data Availability

The raw data supporting the conclusions of this article will be made available by the authors, without undue reservation.

## Appendix 1

Measurement items. Group music activity participation GMAP1. I actively participate in group music activities. GMAP2. I work with my classmates to ensure that music activities go smoothly. GMAP3. I contribute positively to group rehearsals and performances. GMAP4. I am fully engaged during collaborative music activities. Musical self-expression MSE1. I use music to communicate feelings that cannot be expressed verbally. MSE2. Music allows me to express my personal identity. MSE3. I feel that music reflects my inner emotions. MSE4. I express myself freely when participating in music activities. Musical empathy ME1. I feel emotionally connected to others while we play or sing together. ME2. I can sense the emotions of my fellow musicians during performance. ME3. I understand the feelings of others through shared musical experiences. ME4. I adjust my performance based on the emotional cues of other group members. Pro-social behavior PSB1. In an emergency, I do not hesitate to help others. PSB2. I willingly share my resources with others when needed. PSB3. I try to comfort people who are upset. PSB4. I cooperate with others to solve problems.
